# Non-Bulk Morphologies of Extremely Thin Block Copolymer Films Cast on Topographically Defined Substrates Featuring Deep Trenches: The Importance of Lateral Confinement

**DOI:** 10.3390/polym15041035

**Published:** 2023-02-19

**Authors:** Elisheva Michman, Meirav Oded, Roy Shenhar

**Affiliations:** Institute of Chemistry and the Center for Nanoscience and Nanotechnology, The Hebrew University of Jerusalem, Jerusalem 9190401, Israel

**Keywords:** block copolymers, directed self-assembly, thin films, hierarchical structures, patterning

## Abstract

Directed self-assembly of block copolymers is evolving toward applications that are more defect-tolerant but still require high morphological control and could benefit from simple, inexpensive fabrication processes. Previously, we demonstrated that simply casting ultra-thin block copolymer films on topographically defined substrates leads to hierarchical structures with dual patterns in a controlled manner and unraveled the dependence of the local morphology on the topographic feature dimensions. In this article, we discuss the extreme of the ultraconfined thickness regime at the border of film dewetting. Additional non-bulk morphologies are observed at this extreme, which further elaborate the arsenal of dual patterns that could be obtained in coexistence with full placement control. It is shown that as the thickness confinement approaches its limit, lateral confinement imposed by the width of the plateaus becomes a critical factor influencing the local morphology.

## 1. Introduction

Block copolymers (BCPs) are macromolecules that contain two or more distinct polymer chains that are covalently bonded to one another. BCPs undergo microphase separation, forming arrays of nanostructures such as lamellae, cylinders, and spheres [1,2]. Thin films of microphase-separated BCPs have been used to create stripe or dot patterns for a number of applications [3,4,5,6]. Commonly, patterned BCP thin films are used as etch masks in pattern transfer steps of semiconductor materials [7,8,9,10], as well as to organize nanoparticles [11,12,13,14,15,16,17,18] or functional materials [19,20], for nanoporous filtration membranes [21,22,23,24,25], nanotextured surfaces [26], and biomedical devices [27,28].

Domain orientation can be controlled by a process of directed self-assembly (DSA), where pre-patterning of the substrate is used to guide the self-assembly of the BCP during microphase separation [5,29,30,31,32]. DSA is one of the most promising approaches for high-end applications requiring oriented nano-domains. To this end, research in the field of DSA of BCPs for nanofabrication applications has centered on achieving highly ordered periodic features [5,33,34,35,36,37,38,39,40,41] and programmable design control for defect-intolerant applications such as integrated circuits [10,42,43,44,45,46,47,48,49,50,51,52,53,54,55,56,57]. The prerequisites to these applications are the ability to obtain complex patterns, exacting control over feature size, and reproducibility [58,59,60,61,62,63,64]. Addressing these demands often requires prepatterning nanofabrication stages [65], usually performed by chemoepitaxy [35,39,66,67,68], topographical patterning [16,37,38,42,61,69], or in combination with other techniques [5,65,70].

The exacting requirements necessary for defect-free nanofabrication usually lead to a complex and expensive fabrication process. Recently, efforts have been directed toward broadening the scope of DSA of BCPs to applications that are less stringent in their requirement for defect-free structures but still require a moderate-to-high level of ordering and placement control [6] and could be realized by simple and inexpensive fabrication processes. We have recently demonstrated a simple fabrication process that capitalizes on the combination of substrate topography with thickness confinement to effect the formation of co-existing, dual patterns, where the location of each pattern is dictated in a fully controlled manner by the pre-designed location of the topographic feature [71]. This behavior was rationalized by the topography-driven variation in film thickness between the film coating the plateaus and the film deposited in the trenches, which is coupled to the extreme sensitivity of the morphology to the local film thickness for films confined to thicknesses lower than half the lamellar period [72,73]. In a follow-up study, we have unraveled the intricate relationship between the trench depth, lateral topographical dimensions, and film surface profile, allowing us to predict the morphology of the film under various experimental conditions while the nominal film thickness remained constant [74].

The current work expands the fundamental exploration of the morphological behavior of ultraconfined films cast on topographically defined substrates to the boundary of dewetting. As will be shown below, additional co-existing, non-bulk morphologies are obtained at this limit, and a new dependence on the lateral dimensions of the plateaus and trenches emerges.

## 2. Materials and Methods

PS-*b*-PMMA diblock copolymer (*M*_n_ 312 kDa, PDI 1.27, 45 wt% PS, *L*_0_ = 84 nm) was synthesized by standard anionic polymerization under a nitrogen atmosphere. The molecular weight, size distribution, and polystyrene weight percentage were determined by gel permeation chromatography (GPC) in tetrahydrofuran against PS standards for the PS block and by comparison of the ^1^H NMR signals for the phenyl and methoxy groups for the PMMA block. *L*_0_ was determined by SAXS.

Topographically patterned substrates were prepared by etching 38 nm-deep trenches with varying widths into polished silicon wafers (~0.25 nm roughness) coated with a native oxide layer. Substrates were coated with a 250 nm-thick PMMA resist (495 kDa, Microchem), and the features were defined using electron beam lithography (Raith e-LINE), followed by cold development (2 min, −5 °C) in MIBK:IPA (1:3) developer solution and reactive ion etching with C_4_F_8_ and SF_6_ (Oxford Instruments Plasmalab System 100). The topographically defined substrates were subsequently cleaned by oxygen plasma and piranha solution. Trench depth measurements were performed using scanning force microscopy (SFM; see Figure S1).

Block copolymer thin films were prepared on patterned substrates by spin coating toluene solutions of BCP of various concentrations (0.3% to 1% *w*/*w*) for 30 s at 3000 rpm. All films were annealed for 15 min in a closed petri dish with a saturated chloroform vapor environment at ambient temperature.

Film characterization was performed using a high-resolution scanning electron microscope (FEI Sirion HR-SEM) and scanning force microscopy (SFM, Dimension 3100 or Dimension Icon XR, Bruker). Film thicknesses above the trenches and plateaus were determined by scratching away part of the film with a 19-gauge syringe needle, followed by SFM scanning and analysis of the seam between the intact BCP film and the exposed silicon substrate using the step analysis tool (Nanoscope Analysis Program v. 1.40 and 2.0, Bruker), which averages height values of different scan lines of selected areas. These thicknesses were determined by referencing the measured heights to the SFM height values of the corresponding, completely exposed features (see Figure S2) [71]. Film height contrast was measured using step analysis from the highest area of the plateau to the lowest area of the trench [74]. For films where the substrate became exposed by dewetting (i.e., BCP droplets), cross-section analysis was used to measure the film thickness at the desired location. At least three height boundaries on two separate scans were measured for each plateau width, and an average value was used for the data plot.

## 3. Results and Discussion

Lamellar polystyrene-*block*-poly(methyl methacrylate) (PS-*b*-PMMA) diblock copolymer (*M*_n_ 312 kDa, PDI 1.27, *f*_PS_ = 0.48, *L*_0_ = 84 nm) was spin coated from toluene solutions over topographically patterned substrates yielding films with nominal film thicknesses, *h*, in the range of 8–24 nm. The high limit of this thickness range overlaps with the nominal film thicknesses studied in our previous works (22–23 nm) [71,74], which are already considered ultra-confined (i.e., *h* < 0.5 *L*_0_), whereas the other film thicknesses extend the investigations to even stronger confinement. The topographically patterned substrates were made of silicon wafers that were etched with an array of 38 nm-deep, parallel trenches (the trench depth was kept constant throughout this study). In contrast to our previous investigations, this trench depth was larger than all the nominal film thicknesses explored in this study. The plateau widths varied in the range of 160 to 640 nm (~2–8 *L*_0_), and the trench widths varied in the range of 160 to 2000 nm. After spin coating, the films were annealed in saturated chloroform vapor for 15 min at ambient temperature. Chloroform was selected for solvent annealing because it is rather nonselective toward PS and PMMA (χ_CHCl3,PS_ and χ_CHCl3,PMMA_ values calculated using Hansen solubility parameters equal to 0.47 and 0.50, respectively) [75].

**Figure 1:**
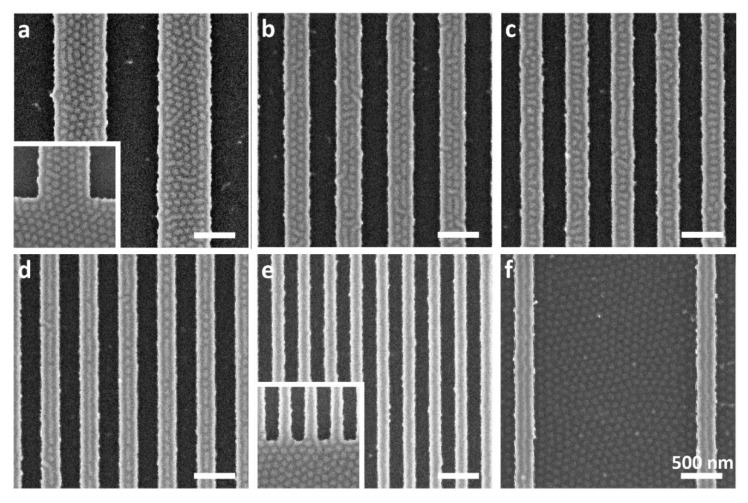
SEM images of samples with nominal 18 nm-thick BCP film annealed over a topographically patterned substrate featuring parallel plateaus and trenches (light and dark tones, respectively) with 38 nm deep trenches and variable trench/plateau widths: (**a**) 640/640 nm, (**b**) 320/320 nm, (**c**) 280/280 nm, (**d**) 240/240 nm, (**e**) 160/160 nm, and (**f**) 2000/240 nm. Insets in (**a**,**e**) show the edge of the patterned area. White domains (dots and stripes) correspond to PMMA. All scale bars represent 500 nm.

Figure 1 shows scanning electron microscopy (SEM) images of an 18 nm nominal film thickness film. No surface pattern is visible in the trenches that are 640 nm-wide or less (Figure 1a–e). The local film thicknesses in the trenches were measured in the range of 20 to 25 nm (see the Experimental section for additional details), which, in accordance with our previous work, yields a lying lamellar morphology [71]. The films on the plateaus are much thinner, in the range of 9 to 14 nm. In this film thickness range, the surface pattern on the plateaus is primarily dependent on the plateau width. For wide plateaus, dots are visible over the central area of the plateau; striped domains are observed close to the edge of the plateau (Figure 1a). As will be shown below, these stripes are not associated with charging artifacts, but represent PMMA domains. A mixture of co-existing patterns, namely dots and stripes (oriented both parallel and perpendicular to the plateau), is visible on 280 and 320 nm wide plateaus (Figure 1b,c). For plateau widths of 240 nm or less, a stripe oriented parallel to the topographic features is also seen at the center of the plateau (Figure 1d). It is interesting to note that while the surface patterns exhibited by thicker films (22–23 nm nominal thickness) cast over shallower trenches, which were described in our previous study, showed a trivial dependence on the plateau width (i.e., the width merely dictated the number of dots across the plateau) [74], the 18 nm-thick films investigated in this study exhibited a different morphology on the narrowest plateaus. This observation shows that increasing the ratio between trench depth and film thickness and utilizing narrow plateaus provides access to non-bulk patterns of co-existing domain types that have not been observed before.

Films cast on substrates featuring wider trenches (i.e., a sparse topographic array) reveal a dot pattern in the trenches (Figure 1f). This observation indicates that the resulting film in wide trenches is thinner than in films cast on denser arrays of topographic features, which is corroborated by the known tendency of the latter to retain more material during the spin coating process [76,77]. Indeed, scanning force microscopy (SFM) measurements show that the average film thickness in trenches that are at least 1 μm-wide is 15.7 ± 4.3 nm, nearly 7 nm less than that of the trenches that are narrower than 1 μm (average film thickness: 22.6 ± 1.3 nm). In contrast, the average thickness of films on the plateaus in these cases differs by only 2 nm (9.9 ± 1.6 and 12.0 ± 1.4 nm, respectively). The strong dependence of the film thickness in the trenches on trench width and the weak dependence of the film thickness on the plateaus on trench width are explained by the considerably less hindered motion of the solution across the plateaus during spin coating compared with its motion in the trenches, where it is restricted by the trench walls. Hence, as dense arrays retain more material than sparse arrays during spin coating, variation in trench width would mostly influence the amount of material retained in the trenches than the amount of polymer deposited on the plateaus, leading to less deposited polymer in wide trenches than in narrow trenches (whereas the amount of polymer deposited on the plateaus is rather indifferent to the width of the trenches). A similar conclusion may be drawn by considering that wide trenches present more surface area of floor and walls with respect to the surface area of the narrow plateaus, and that the walls are expected to be slightly rougher than the polished surfaces of the plateaus (which is known to influence polymer dynamics [78,79]). Both effects promote the adhesion of more material to the trenches during spin coating. Therefore, the amount of polymer deposited in the trenches is expected to depend more strongly on the trench width than the amount of polymer deposited on the plateaus.

**Figure 2:**
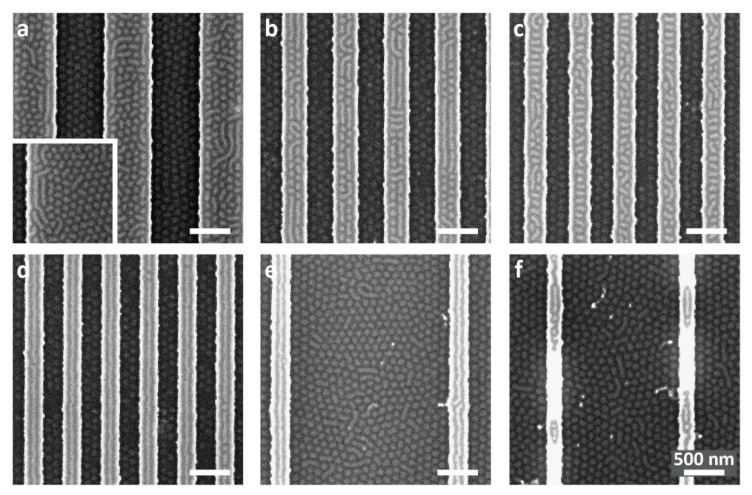
SEM images of samples with nominal 15 nm-thick BCP film annealed over a topographically patterned substrate featuring 38 nm deep trenches and variable trench/plateau widths: (**a**) 600/600 nm, (**b**) 320/320 nm, (**c**) 280/280 nm, (**d**) 240/240 nm, (**e**) 2000/240 nm, and (**f**) 1500/160 nm. Inset in (**a**) shows the edge of the patterned area. All scale bars represent 500 nm.

Figure 2 shows SEM images for samples featuring a thinner, 15 nm nominal film thickness cast over the same topographic array. The slightly thinner film gives rise to the appearance of dots in the trenches, independent of the trench dimensions. The films on the plateaus exhibit similar morphologies as the samples shown in Figure 1 above, with some noteworthy differences. When dots coexist with parallel stripes on the plateaus, the fraction of stripes is larger for the sample with the thinner nominal film (compare Figure 2a to Figure 1a and Figure 2b to Figure 1b). Additionally, the middle stripes of the 3-stripe pattern on the plateaus in Figure 2d are nearly complete for the 15 nm nominal film thickness, whereas in the slightly thicker (18 nm) film they are broken into stripe segments and even dots (Figure 1d).

The differences observed in the local morphologies on the plateaus and their dependence on slight variations in film thickness are explained as follows. Within the ultra-thin confinement regime, three types of morphologies were identified by computer simulations [71]. At the low film thickness limit within this regime, the films exhibit a striped pattern because orienting the PS/PMMA lamellae normal to the substrate minimizes the PS/PMMA interfacial area per chain. Conversely, at the high film thickness limit within the ultra-thin confinement regime, where thickness allows the accommodation of an average normal orientation of the chains, orienting the domains parallel to the substrate yields a lower interfacial area. This phenomenon is particularly pronounced for highly selective substrates. In between these limits, for intermediary substrate selectivity and film thickness, which correspond to the BCP and substrate used in this study, a dot pattern appears. This pattern is the manifestation on the free surface of the film of a non-bulk, neck-like morphology, where the more substrate-compatible PMMA domains create the widened bases of the neck-like structure. Thus, when the local film thickness on the plateaus is close to the transition from stripes to dots, co-existing patterns are observed on the plateaus (as is evident with the 15 nm nominal thickness films).

For samples featuring plateaus of 240 nm or less, the films on the plateaus exhibit a pattern of only stripes, which are oriented parallel to the plateau direction (Figure 2d,e). The ability to influence the pattern on the plateaus using the plateau width and obtain contrasting, distinct patterns on the plateaus and in the trenches (stripes and dots, respectively) manifests the control provided by substrate topography on obtaining not only dual, coexisting patterns but also dictating the boundaries between them using simple preparation steps.

**Figure 3:**
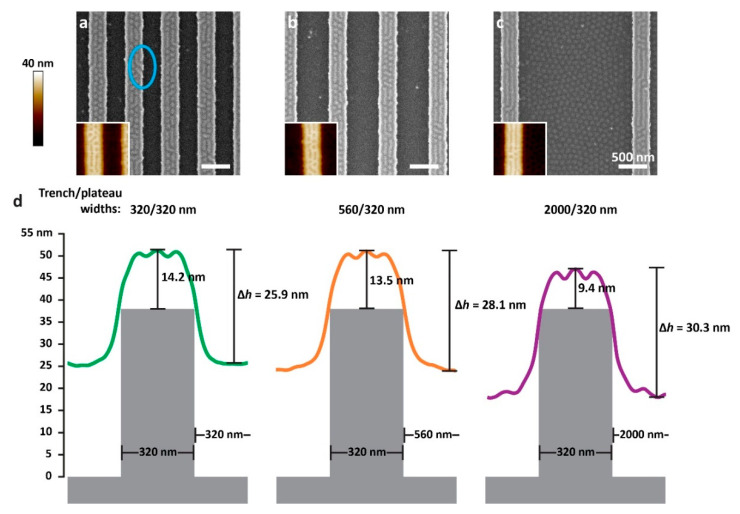
SEM images of samples of 18 nm-thick films cast over substrates with 38 nm-deep trenches and lateral trench/plateau widths of: (**a**) 320/320 nm, (**b**) 560/320 nm, and (**c**) 2000/320 nm. All scale bars represent 500 nm. Insets in panels a-c show SFM height images of the corresponding sample (identical magnification as the SEM images). Blue ellipse indicates defects in the stripe at the right-hand edge of the plateau. (**d**) An illustration showing the experimental height cross-section corresponding to each of the SEM images overlaid on schematics of the substrate topography. The height of the film over the plateau was established according to local film measurements for these samples.

Even though the pattern on the plateaus seems to be primarily dependent on the plateau width, a comparison of samples with the same plateau width and different trench widths reveals a mild dependence on the trench width as well. Figure 3 shows the patterns observed on three arrays featuring the same plateau width (320 nm) and varying trench widths (320, 560, and 2000 nm). In all cases, the patterns on the plateaus consist of co-existing dots and stripes, where the composition gradually changes from dots to stripes as the trench separating adjacent plateaus becomes wider. As shown in Figure 3d, as the trench width increases, the film becomes locally thinner both on the plateau and in the trench. Consequently, according to the arguments mentioned above [71], the fraction of dot structures on the plateau decreases and the fraction of stripes increases.

Although the films are continuous and do not dewet from the sidewalls of the trenches (possibly also because of the slightly increased roughness of the sidewalls compared with the horizontal surfaces [78,79]), the overlays shown in Figure 3d indicate that the surface of the film, regardless of trench width, is extremely close to the edge of the plateau, which suggests pinning of the film to the plateau edges. The formation of stripes at the edges of all of the plateaus shown in Figure 1 and Figure 2 (which were not observed with the thicker films in our previous studies [74]) may be templated by such pinning.

**Figure 4:**
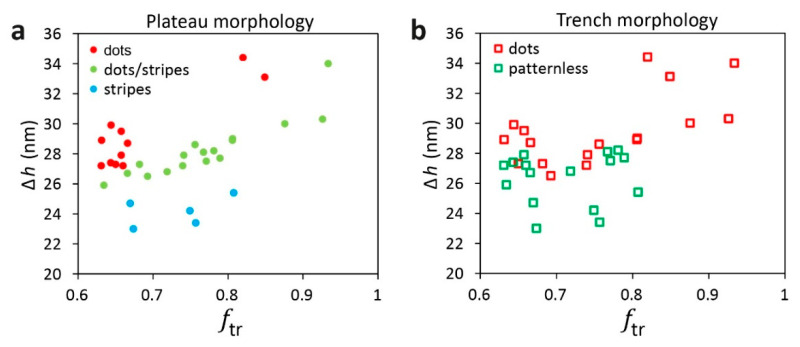
Phase diagrams of the morphologies of lamellar PS-*b*-PMMA cast over topographically patterned substrates with 38 nm-deep trenches: (**a**) patterns that develop on the plateaus; (**b**) patterns that develop in the trenches.

In our previous study [74], we presented phase diagrams that relate the morphologies on the plateaus and in the trenches to the film’s height contrast, Δ*h*, and the fraction of the film that resides in the trench, $f_{\mathrm{tr}}$. The height contrast is defined as the difference between the highest part of the film deposited on the plateau and the lowest part of the surface of the film deposited in the trench, which is directly measured from the SFM images (as shown schematically in Figure 3d). The fraction of the film that resides in the trench is approximated from the lateral dimensions of the topographic features and the local film thicknesses measured by SFM (see Experimental Section and supplementary Figure S2 for additional details) according to the following equation:

(1) $$f_{\mathrm{tr}}=\frac{h_{\mathrm{tr}}w_{\mathrm{tr}}}{h_{\mathrm{tr}}w_{\mathrm{tr}}+h_{\mathrm{pl}}w_{\mathrm{pl}}}$$

where *h*_tr_ and *h*_pl_ are the local film thicknesses in the trenches and on the plateaus, respectively, and *w*_tr_ and *w*_pl_ are the respective widths of the trench and plateau. Figure 4 shows phase diagrams constructed with the data shown in this paper.

The choice of Δ*h* and $f_{\mathrm{tr}}$ as the variables for the phase diagrams is rationalized as follows. The height contrast correlates with the steepness of the slope of the film profile across the edge of the plateau. A mild slope indicates a gradual change in the film thickness near the edge of the plateau, which contradicts the natural tendency of block copolymer films to favor discrete film thicknesses. As we have argued in our previous study [74], the conformational frustration caused to the polymer chains by this situation may lead to nucleation of perpendicularly-oriented PMMA domains at the edges of the plateaus [3,80], which further template the formation of additional domains, which propagates toward the center of the plateaus. The observation of the stripes at the edges of the plateaus in this study seems to corroborate this explanation. Whereas the vertical coordinate of the phase diagram relates to the film profile, the horizontal coordinate relates to the dimensions of the topographic features of the substrate, which have been shown to influence the film profile [76,77]. The choice of $f_{\mathrm{tr}}$ among other possible variables that relate to the substrate topography (such as duty cycle or feature density) was made because it provided the best separation between different morphologies, which emphasized the need to include the local film thicknesses in this variable.

The resulting phase diagrams, based on Δ*h* and $f_{\mathrm{tr}}$, tie the morphologies that develop on the plateaus and in the trenches to the film profile, local film thicknesses, and the topographic features dimensions. Yet, the need to know Δ*h* and $f_{\mathrm{tr}}$ for a given polymer system hinders the ability to predict the local morphologies from the given dimensions of the topographic features. Fortunately, we have shown in the previous study that both variables exhibit simple relationships to the trench depth, *d*, and the widths of the plateaus and trenches (*w*_pl_ and *w*_tr_). When applied to films featuring the same nominal film thicknesses cast over topographic substrates with different feature depths, we obtained the following equation (where all variables are given in nm) [74]:

(2) $$\Delta h=\left(0.8140-\frac{93.44}{w_{\mathrm{tr}}+w_{\mathrm{pl}}}\right)d$$

**Figure 5:**
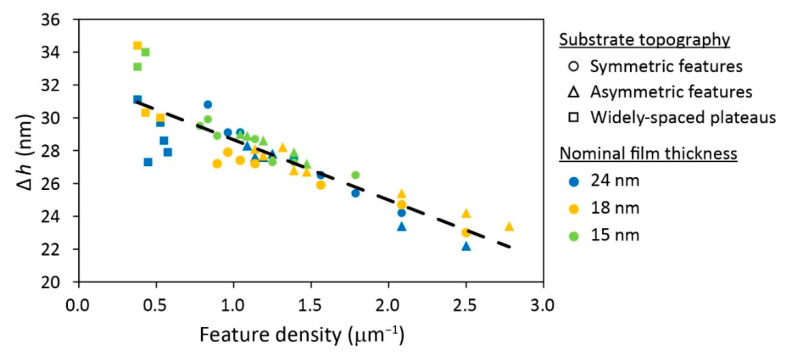
Dependence of height contrast on the topographic feature density for samples with 38 nm trench depth.

The same analysis was now applied to the data discussed in the current study, where the trench depth was fixed at *d* = 38 nm and the nominal film thickness was varied. Figure 5 shows the dependence of the experimentally measured height contrast on the feature density, (*w*_pl_ + *w*_tr_)^−1^, for different types of arrays: symmetric (i.e., *w*_pl_ = *w*_tr_), asymmetric (i.e., *w*_pl_ < *w*_tr_), and largely asymmetric (i.e., where the plateaus are spaced by very wide trenches). In accord with our previous results, the data, which were obtained from the data on the 18 and 15 nm-thick films combined with the data previously reported on 24 nm-thick films cast on the same substrates [74], show a linear relationship between the height contrast and the feature density, which is found to be independent of the nominal film thickness (at least for the range that was investigated thus far):

(3) $$\Delta h=32.32-\frac{3669}{w_{\mathrm{tr}}+w_{\mathrm{pl}}}$$

This equation, which was mostly derived from the new data presented in this article, agrees within 5% deviation with equation 2, enables to estimate the expected value of Δ*h* from the widths of the plateaus and trenches and thus facilitates the prediction of the local morphologies from the phase diagrams before performing the experiment.

A similar analysis yielded the following dependence of $f_{\mathrm{tr}}$ on the duty cycle (defined as *w*_pl_/(*w*_pl_ + *w*_tr_)) and the trench depth for films featuring nominal film thickness of 22–23 nm [74]:

(4) $$f_{\mathrm{tr}}=1+\left(0.005672d-0.9602\right)\frac{w_{\mathrm{pl}}}{w_{\mathrm{pl}}+w_{\mathrm{tr}}}$$

**Figure 6:**
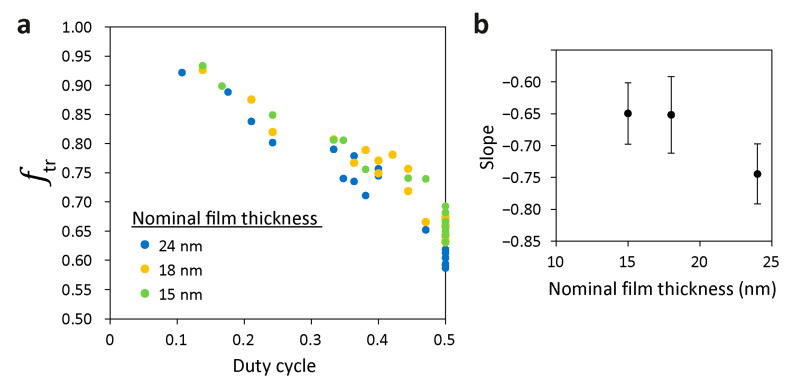
(**a**) Dependence of the fraction of BCP in the trench on the duty cycle (see supplementary Figure S3 for the individual curves). (**b**) Dependence of the slopes of the data in (**a**) on nominal film thickness for a constant trench depth of 38 nm.

The data we now have on thinner films (15 and 18 nm) cast on topographic substrates with *d* = 38 nm show a somewhat weaker dependence of $f_{\mathrm{tr}}$ on the duty cycle (i.e., smaller negative slopes than the ones observed for 22–23 nm-thick films; see Figure 6 and Figure S3). At the regime of extreme confinement, this relationship seems independent of film thickness. Nonetheless, the difference of ~15% between the slopes calculated from the data on the films in the extreme confinement regime (*h*_nominal_ ≤ 18 nm) and the slope calculated for the 22–23 nm-thick films hint that there might be two distinguished thickness regimes. Yet, additional data on films cast at additional film thicknesses in 38 nm-deep trenches as well as data on films with variable film thicknesses cast on substrates featuring other trench depths are required to substantiate the influence of the nominal film thickness on $f_{\mathrm{tr}}$.

We observed that the thinnest films (15 nm nominal thickness) exhibit partial dewetting when cast on narrow plateaus that are separated by wide trenches (Figure 2f). As discussed above, topographic patterns featuring widely spaced plateaus retain less material on the substrate during spin coating. Additionally, relatively deep trenches direct a larger fraction of the material into the trench by capillary force. The combination of both effects depletes the amount of BCP that remains on the plateau after spin coating. Below a critical thickness that depends on the surface energies of the polymer and the substrate, the film dewets from the surface of the plateau.

Generally, the dewetting of films is difficult to control, hence it is not a desirable phenomenon in directed self-assembly of block copolymers [81]. However, the employment of topographic substrates provide a way to control the location of the occurrence of dewet regions and thus confine the unique patterns formed by dewetting to the plateaus. This allows not only to obtain complex, non-bulk patterns in a controlled fashion but also to unravel the behavior of the BCP film in the extreme confinement regime.

**Figure 7:**
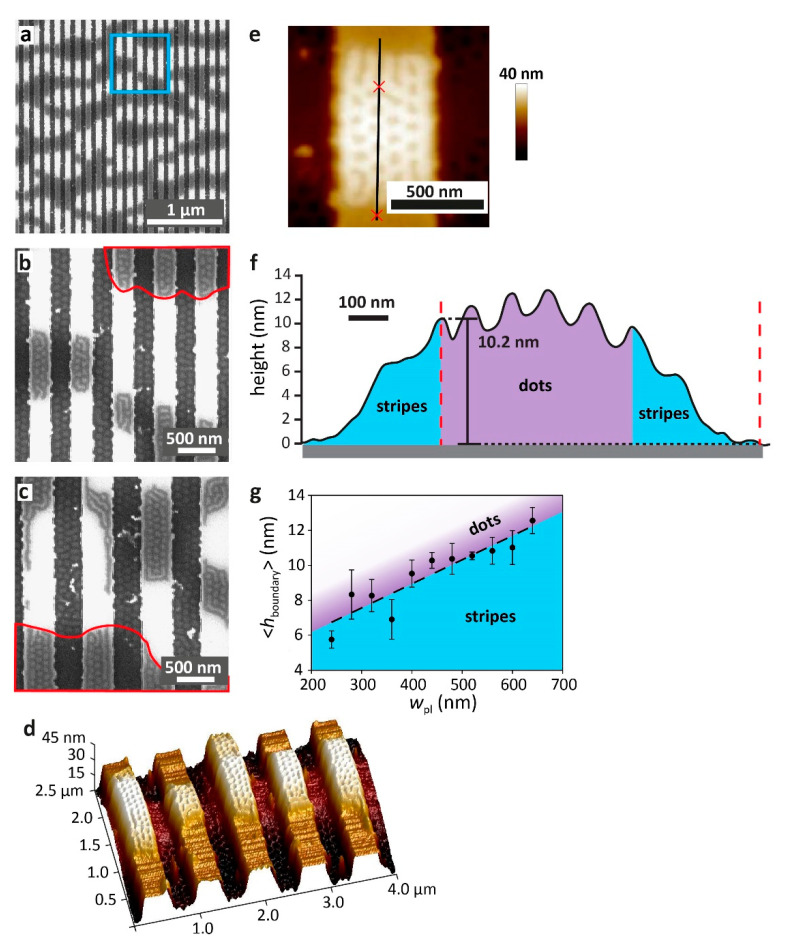
(**a**–**c**) SEM images of an ultrathin, 8 nm-thick film cast over topographically defined substrates featuring 38 nm-deep trenches. Bright white areas correspond to exposed regions of the plateaus. Lateral dimensions of trench and plateau are 280 (**a**,**b**) and 400 nm (**c**). (**b**) High magnification image of the blue boxed area in (**a**). Red enclosed areas in (**b**,**c**) demark film regions that are thicker than the adjacent regions on both the plateau and in the trench. (**d**) Three-dimensional SFM height image of the sample shown in (**a**). (**e**,**f**) SFM image and corresponding cross-section of a typical dewet area on the plateau, showing the transition (from top to bottom in (**e**)) from a pattern of dots at the center of the dewet area through a pattern of stripes to the exposed plateau surface. (**g**) Graph of the local film height at the boundary where the dot pattern transitions to a stripe pattern. Error bars represent the standard deviation in the measurements; the dashed line represents the linear regression. Blue and purple shaded regions in the graph denote the ranges of local film thicknesses where stripes and dots are observed, respectively.

Figure 7 shows samples with a nominal film thickness of 8 nm. Interestingly, upon annealing, the film dewet over the patterned area but remained intact in unpatterned regions (see supplementary Figure S4). Figure 7a,b show an area of dewet film cast on a substrate featuring 280 nm-wide plateaus and trenches. White areas correspond to exposed regions of the plateaus, from which the polymer dewet. We noticed that the dewetting pattern is oriented perpendicular to the direction of the topographic features. This dewetting behavior is common to all the patterned areas studied in this work. Previous research using polystyrene-*block*-poly(2-vinyl pyridine) (PS-*b*-P2VP) showed the formation of islands and holes, which were structured similarly to the dewet domains in this study (i.e., islands developed almost exclusively over the topographically patterned region of the substrate and were elongated perpendicular to the direction of the topographic features) [82]. It was concluded that the local thickness incommensurability that is inherently induced by the topographic pattern led to the formation of islands and holes. Additionally, consideration of the forces acting on the polymer film while it coarsens during annealing led to the conclusion that the flow of material occurs mostly along the trenches and plateaus in a correlated motion, and thus the islands that form appear elongated perpendicular to the direction of the topographic features and continue smoothly across the plateau edge [82]. It seems reasonable to assume that the dewetting of ultrathin PS-*b*-PMMA films on deep topographic patterns, which is analogous to the formation of islands and holes, occurs in a similar fashion.

Figure 7d shows a three-dimensional SFM height image of the same sample, featuring 280 nm-wide trenches and plateaus. Although the film formed dewet domains and regions with exposed plateau surfaces, the film did not dewet in the trenches but rather coarsened during the annealing process, forming islands and holes along the trench direction. Analogously to the correlated coarsening of PS-*b*-P2VP films [82], a spatial correlation is observed between the terracing patterns in the trenches and the dewetting patterns on the plateaus. Figure 7b,c, which show high-magnification SEM images of dewet films cast over different patterns, reveal that trench regions that are flanked by exposed plateau regions on both sides exhibit a dot morphology, which is consistent with the lower film thickness observed in these regions (Figure 7d). Trench regions that are flanked by plateau regions covered by the polymer appear patternless, which is consistent with the higher film thickness in these regions (see the red enclosed areas in Figure 7b,c). It seems that as the film on the plateau recedes along the plateau direction, exposing the plateau surface, the material in the trench recedes with it along the trench direction.

The polymer droplets on the plateaus are also phase-separated, showing co-existing stripes and dots (see supplementary Figure S5). The stripes are closest to the edge of the polymer droplets, where the film gradually thins toward the exposed region. The formation of standing lamellae at this gradient thickness region is explained by the interfacial area consideration described above. The thickness of the PS domains, *h*_boundary_, at the grain boundary separating the PMMA dots region and the margin of the droplet, where the film gradually thins and shows stripes, was measured as shown in Figure 7e,f for various samples featuring symmetric topographic patterns (*w*_pl_ = *w*_tr_) with varying plateau/trench widths. Interestingly, we find that *h*_boundary_ correlates linearly with the width of the plateau (Figure 7g), which means that the minimum thickness required for the formation of the dot pattern increases with increasing plateau width. This observation expands our understanding of ultraconfined films [65,71,72,74,83] and shows that the morphology is influenced not only by the thickness confinement but also by the lateral confinement. Hence, *h*_boundary_ may serve as a quantitative manifestation of the lateral confinement effect.

## 4. Conclusions

This work focuses on the effect of the lateral dimensions of the trench and plateaus on the directed self-assembly of ultraconfined block copolymer films at the limit of low film thickness toward dewetting. In addition to the dependence of the BCP surface pattern on the film thickness and the trench depth [71,74], the morphology of extremely thickness-confined films is highly sensitive to the lateral confinement imposed by the widths of the plateaus and trenches. Under the right conditions, new combinations of co-existing morphologies can be achieved. Whereas the nominal film thickness and the trench depth cannot be easily varied on a single sample, the lateral dimensions of the topographic pattern can be designed at will to achieve the desired patterns, including spaced regions of one pattern (e.g., dots and/or stripes) separated by regions of another pattern (e.g., non-patterned or dots). Additionally, the spacing of the topographic features allows some control over the local film thickness. Although the morphologies in this study were demonstrated on topographic patterns composed of straight plateaus, irregular topographic patterns can be used with equal success [71].

Examination of the morphologies of dewet polymer droplets on the plateaus unraveled an unexpected dependence on the minimal film thickness required for the formation of a dot morphology on the plateau width. This finding emphasizes that while the local film thickness is the dominant factor dictating the morphology in the ultraconfined regime (where the film thickness is lower than 0.5 *L*_0_), lateral confinement becomes an influential factor at the extreme of low film thickness (toward dewetting) and adds to the considerations that have to be taken into account. In non-dewet films, this insight explains the formation of a stripe pattern on the narrower plateaus and a dot pattern on the wider (i.e., less confined) plateaus.

We expect that the ability to obtain co-existing patterns with precise locations over the substrate by harnessing the combination of thickness confinement with the thickness differentiation and lateral confinement afforded by topography will promote the development of advanced photonic devices [6].

## Data Availability

Not applicable.

## Supplementary Materials

The following supporting information can be downloaded at: https://www.mdpi.com/article/10.3390/polym15041035/s1**,** Figure S1: SEM image (a), SFM height image (b), and SFM height profile (c) for a bare silicon substrate after patterning and cleaning. Substrate dimensions are trench depth, 38 nm; trench/plateau widths, 640/640 nm. Figure S2: SFM height image (a) and height profile (b) of an area that was partially exposed by film scratching (nominal film thickness, 15 nm; trench depth, 38 nm; trench/plateau widths, 640/320 nm) showing the method of local film thickness analysis. The height profile in (b) is overlaid on a schematic illustration of the substrate topography. Figure S3: Dependence of fraction of BCP in the trench on the duty cycle for different nominal film thicknesses: (a) 15 nm; (b) 18 nm; (c) 24 nm. Dashed lines indicate the linear regression results. Figure S4: SEM overview image of 8 nm-thick film cast on a topographic substrate (lateral trench/plateau widths: 2000/640 nm; trench depth: 38 nm). Note that dewetting occurs only on the plateaus. Figure S5: SFM images of phase separated BCP droplets on the plateau areas.
