# Supplementary material for: Non-Bulk Morphologies of Extremely Thin Block Copolymer Films Cast on Topographically Defined Substrates Featuring Deep Trenches: The Importance of Lateral Confinement
Source: Polymers (Basel). 2023 Feb 19;15(4):1035. doi: 10.3390/polym15041035 (PMC9958675; doi:10.3390/polym15041035)
Supplement: Supplementary file 1 [file polymers-15-01035-s001.zip › polymers-2207507-supplementary-done.pdf]

polymers

MDPI

Article

Non-Bulk Morphologies of Extremely Thin Block Copolymer Films Cast on Topographically Defined Substrates Featuring Deep Trenches: The Importance of Lateral Confinement

Elisheva Michman, Meirav Oded and Roy Shenhar *

Institute of Chemistry and the Center for Nanoscience and Nanotechnology, The Hebrew University of Jerusalem, Jerusalem 9190401, Israel; elisheva.michman@mail.huji.ac.il (E.M.); meirav.oded@mail.huji.ac.il (M.O.)
* Correspondence: roys@huji.ac.il

Abstract: Directed self-assembly of block copolymers is evolving toward applications that are more defect-tolerant but still require high morphological control and could benefit from simple, inexpensive fabrication processes. Previously, we demonstrated that simply casting ultra-thin block copolymer films on topographically defined substrates leads to hierarchical structures with dual patterns in a controlled manner and unraveled the dependence of the local morphology on the topographic feature dimensions. In this article, we discuss the extreme of the ultraconfined thickness regime at the border of film dewetting. Additional non-bulk morphologies are observed at this extreme, which further elaborate the arsenal of dual patterns that could be obtained in coexistence with full placement control. It is shown that as the thickness confinement approaches its limit, lateral confinement imposed by the width of the plateaus becomes a critical factor influencing the local morphology.

Keywords: block copolymers; directed self-assembly; thin films; hierarchical structures; patterning

Citation: Michman, E.; Oded, M.; Shenhar, R. Non-Bulk Morphologies of Extremely Thin Block Copolymer Films Cast on Topographically Defined Substrates Featuring Deep Trenches: The Importance of Lateral Confinement. *Polymers* **2023**, *15*, 1035. https://doi.org/10.3390/polym15041035

Academic Editor: Hui Sun

Received: 23 January 2023
Revised: 15 February 2023
Accepted: 16 February 2023
Published: 19 February 2023

Copyright: © 2023 by the authors. Licensee MDPI, Basel, Switzerland. This article is an open access article distributed under the terms and conditions of the Creative Commons Attribution (CC BY) license (https://creativecommons.org/licenses/by/4.0/).

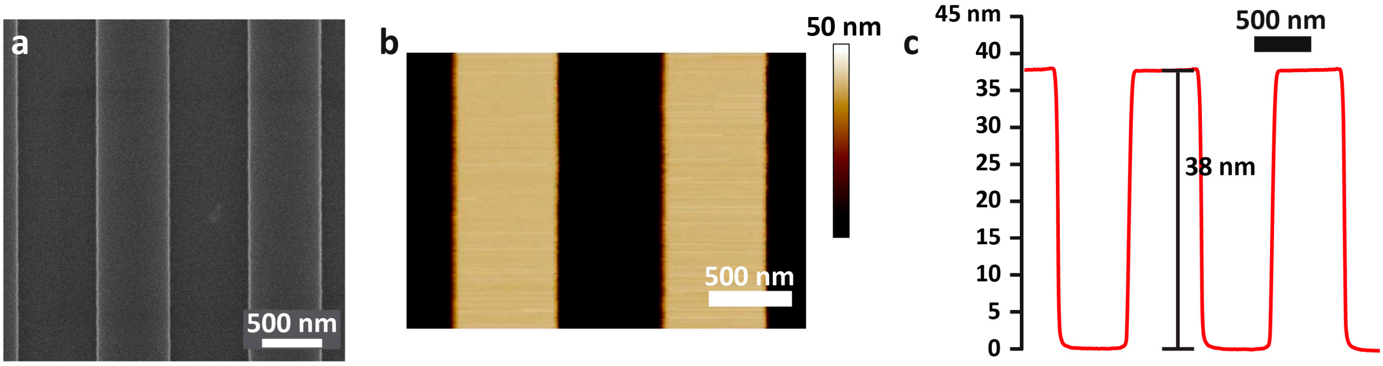

Figure S1. (**a**) SEM image, (**b**) SFM height image, and (**c**) SFM height profile for a bare silicon substrate after patterning and cleaning. Trench depth is 38 nm; trench/plateau widths are 640/640 nm.

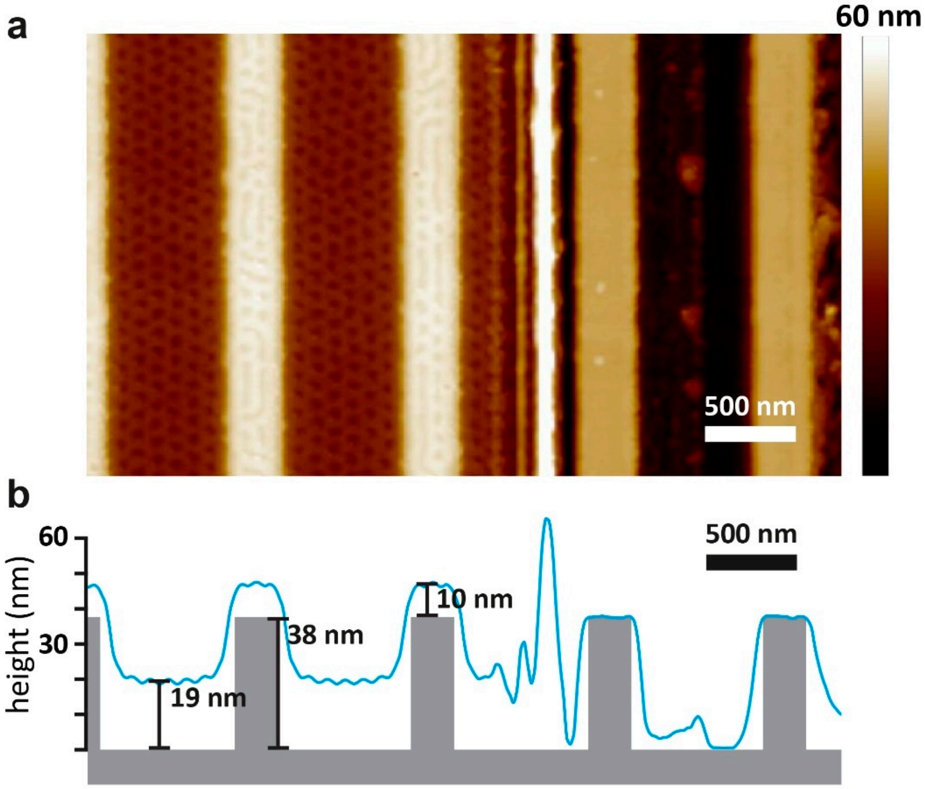

Figure S2. (**a**) SFM height image and (**b**) height profile of an area that was partially exposed by film scratching (nominal film thickness is 15 nm; trench depth is 38 nm; trench/plateau widths are 640/320 nm) showing the method of local film thickness analysis. The height profile in (**b**) is overlaid on a schematic illustration of the substrate topography.

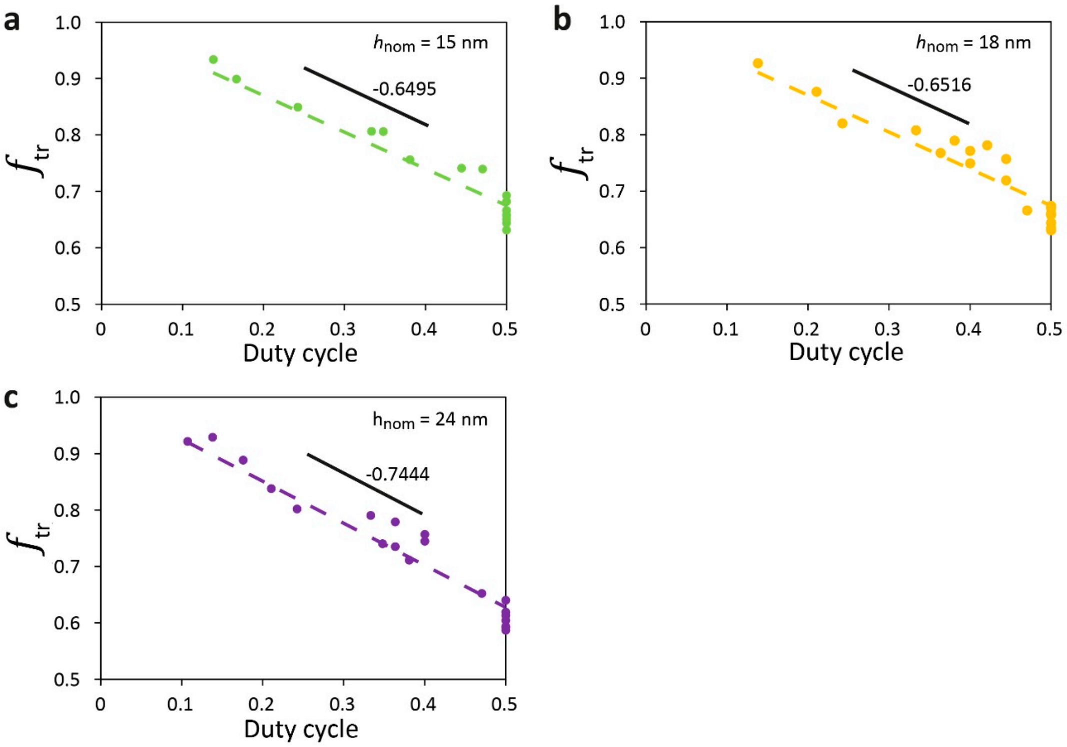

Figure S3. Dependence of fraction of BCP in the trench on the duty cycle for different nominal film thicknesses: (**a**) 15 nm; (**b**) 18 nm; (**c**) 24 nm. Dashed lines indicate the linear regression results.

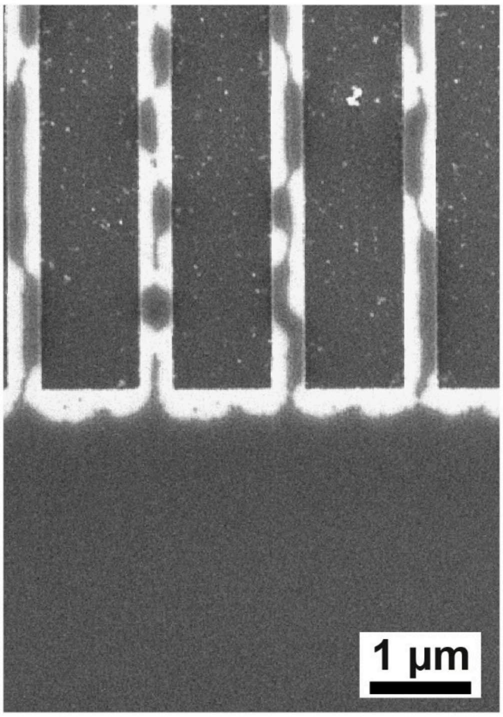

Figure S4. SEM overview image of 8 nm-thick film cast on a topographic substrate (trench depth is 38 nm; trench/plateau widths are 2000/640 nm;). Note that dewetting occurs only on the plateaus.

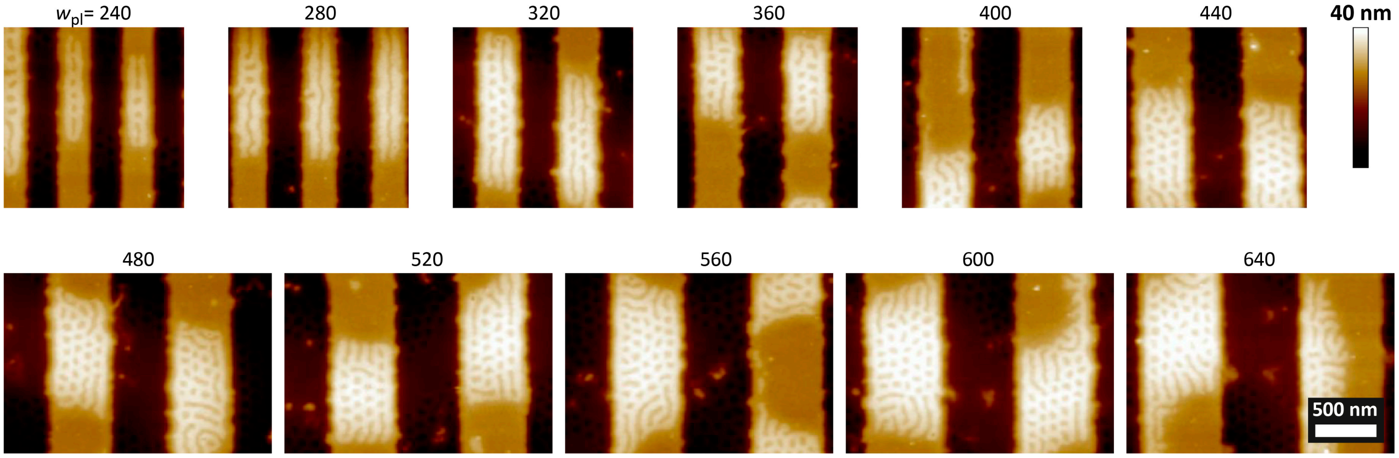

Figure S5. SFM images of microphase-separated BCP droplets on the plateau areas.
